# The cancer testes antigen, HORMAD1, limits genomic instability in cancer cells by protecting stalled replication forks

**DOI:** 10.1016/j.jbc.2023.105348

**Published:** 2023-10-12

**Authors:** Luis Reza Herrera, Ronnesha A. Johnson, Kathleen McGlynn, Zane A. Gibbs, Anthony J. Davis, Angelique W. Whitehurst

**Affiliations:** 1 Department of Pharmacology, UT Southwestern Medical Center, Dallas, Texas, USA; 2 Department of Radiation Oncology, UT Southwestern Medical Center, Dallas, Texas, USA

**Keywords:** cancer testes antigen, HORMAD1, DNA replication stress, genomic instability

## Abstract

Tumors anomalously induce the expression of meiotic genes, which are otherwise restricted only to developing gametes. If and how these aberrantly expressed meiotic proteins influence DNA metabolism is not clear, but could have important implications for how tumors acquire and mitigate genomic instability. HORMAD1 is a highly conserved meiotic protein that is frequently expressed in lung adenocarincoma where its expression correlates with reduced patient survival and increased mutation burden. Here, we find that HORMAD1 associates with the replisome and is critical for protecting stalled DNA replication forks. Loss of HORMAD1 leads to nascent DNA strand degradation, an event which is mediated by the MRE11-DNA2-BLM pathway. We find that these phenotypes are due to limited RAD51 loading onto stalled replication forks in the absence of HORMAD1. Ultimately, loss of HORMAD1 leads to increased DNA breaks and chromosomal defects, which is exacerbated dramatically by induction of replication stress. Tumor cells proliferate despite encountering chronic replication stress, placing them on the precipice of catastrophic genomic damage. Our data support the hypothesis that the aberrant expression of HORMAD1 is engaged to attenuate the accumulation of excessive DNA damage due to chronic replication stress, which may otherwise lead to accumulation of toxic levels of genomic instability.

Cancer testes antigens (CTAs) are a collection of proteins defined by an expression pattern that is normally restricted to reproductive tissues, but also abnormally activated in a wide variety of tumors (1, 2, 3). These proteins are referred to as “antigens” because the testes are an immune privileged site and peptide antigens derived from aberrantly expressed proteins can evoke a patient immune response (4, 5, 6). Thus, CTAs have long been touted as ideal immunotherapeutic targets; an idea that is supported by the success of infusion of patient derived, *ex vivo* expanded T-cells targeting CTAs (7, 8, 9).

Recent studies suggest that CTA proteins are not innocuous when expressed in tumor cells. Instead, a number of reports have identified individual CTAs that are directly engaged in support of neoplastic behaviors, including the following: degradation of tumor suppressors, promoting resistance to apoptosis, enhancing oxidative phosphorylation, and reprogramming transcriptional networks (2, 10, 11, 12, 13, 14, 15, 16, 17). The revelation that these anomalously expressed testes proteins are required for oncogenic behaviors establishes a previously unappreciated aspect of the tumor cell regulatory environment. Moreover, these proteins represent direct intervention targets, which based on their biased expression pattern may have a broad therapeutic window.

Within the CTA family, there are seven proteins with highly conserved functions in meiosis: HORMAD1, HORMAD2, SPO11, SYCE1, SYCE2, SYCP1, SYCP2, and SYCP3 (18, 19, 20, 21, 22, 23, 24, 25, 26). In mammals, these proteins are essential for homologous recombination (HR), which occurs during meiotic prophase 1. Specifically in sperm, HORMAD1 and HORMAD2 are recruited to chromosomes, a process required for the generation of DNA double strand breaks (DSBs) catalyzed by the SPO11 endonuclease (19, 20, 21, 27, 28, 29). After single strand processing, these breaks are the basis for homology search and alignment of chromosomal homologues. Immediately following alignment, the CTAs SYCE1, SYCE2, SYCP1, SYCP2, and SYCP3 promote formation of a proteinaceous bridge between homologous chromosomes termed the synaptonemal complex (22, 23, 25, 26). The synaptonemal complex mediates DSB repair and the formation of crossovers that ultimately permit exchange of maternal and paternal genetic information. Inability to undergo meiotic recombination prevents germ cell maturation. Indeed, deletion of any one of these CTAs in mice leads to infertility in both sexes (18, 19, 20, 22, 23, 24, 25, 26, 30, 31). Notably, these KO mice develop normally without additional detectable defects, indicating the specificity of these CTAs to meiotic processes.

Expression of each of these “meiotic” CTAs has been demonstrated in a wide variety of tumors, and immune reactivity is present in a subset of tumors (4, 32, 33, 34, 35, 36, 37, 38). Given their highly specialized roles in meiosis, it is difficult to predict *ab initio* their function in cancer cells. However, a few details have emerged indicating that these proteins can regulate genomic integrity positively and/or negatively in tumors. Specifically, we and others have found that HORMAD1 is frequently expressed in a wide variety of neoplasms including lung, breast, and ovarian cancers (39, 40, 41, 42, 43, 44, 45). Abnormal expression of HORMAD1 in tumors, like many CTAs, is due to a loss of methylation of its promoter (44). In lung adenocarcinoma (LUAD), suppression of abnormally expressed HORMAD1 attenuates HR repair mechanisms in response to DNA DSBs induced by radiation, poly (ADP-ribose) polymerase (PARP) inhibition, or camptothecin exposure (39, 40, 43, 44). Furthermore, depletion of HORMAD1 attenuates *in vivo* tumor growth in lung cancer xenografts.

In many different tumor types, HORMAD1 expression correlates with increased mutation burden and chromosomal scarring (39, 40, 41, 42, 43, 44, 45). Based on these findings, one question has been whether this correlation is due to HORMAD1 inducing genomic instability or a response to prevent the excessive, and potentially toxic, accumulation of DNA damage. In other words, is HORMAD1 promoting or restraining genetic instability? Here, we report that HORMAD1 protects LUAD tumor cells from genomic instability. Specifically, we find that HORMAD1 associates with the replisome and protects stalled replication forks from excessive MRE11-mediated nucleolytic degradation. Mechanistically, we find that HORMAD1 is required for stabilization of replication fork protection factor, RAD51, onto chromatin. Ultimately, suppression of HORMAD1 in LUAD cancer cells leads to the accumulation of DNA damage and chromosomal aberrations, which is significantly amplified in the presence of replication stress. Our study suggests that anomalously expressed HORMAD1 is engaged in LUAD to cope with replication stress, a chronic source of DNA damage that tumors must mitigate with to prevent catastrophic genomic damage.

## Results

### HORMAD1 protects stalled replication forks from degradation

**Figure 1:**
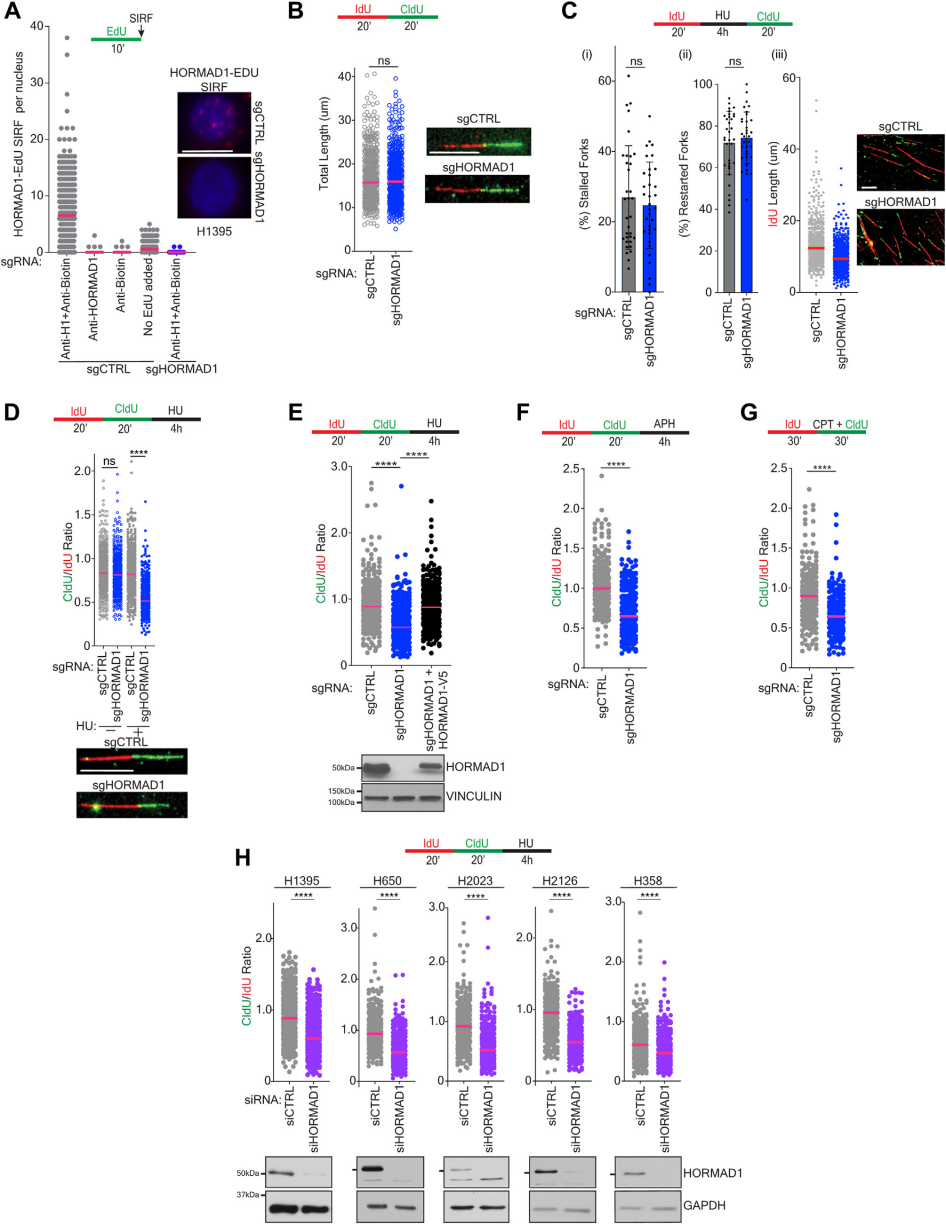
**HORMAD1 protects stalled replication forks from degradation**. *A*, H1395 cells were labeled with 20 μM EdU for 10 min, followed by fixation and SIRF. Each dot represents the number of HORMAD1-EDU foci per nuclei. Mean is indicated. *Left*: images of SIRF signal in individual nuclei. The scale bar represents 10 μm. *B*, DNA fiber assay in H1395 cells according to the schematic (*top*). *Left*: each circle represents the length of individual tracts with median indicated. *p* value calculated by Mann–Whitney test; n = 3*. Right*: representative image of a single DNA fiber. The scale bar represents 10 μm. *C*, DNA fiber assay in H1395 cells according to the schematic (*top*). (i) Bars represent the mean of forks with only IdU labeling as a percentage of all forks observed (error bars represent SEM). *p* value calculated by an unpaired *t* test; n = 3. (ii) Bars represent the mean of tracts labeled with both IdU and CldU as a percentage of total tracts observed (error bars represent SEM). *p* value calculated by an unpaired *t* test; n = 3. (iii) Graph represents the IdU length of individual fibers. Median is indicated. *p* value calculated by Mann-Whitney test; n = 3. *Right*: representative images of fibers are shown. The scale bar represents 10 μm. *D*, H1395 cells were labeled and treated as indicated in schematic (*top*) followed by a DNA fiber assay. Each dot represents the ratio of CldU length to IdU length for an individual tract. Median is indicated. *p* value was calculated by Mann–Whitney test; n =3. *Below*: Representative images of fibers are shown, the scale bar represents 10 μm. *E*, *top*: As in (*D*). *Bottom*: Parallel lysates were obtained, and an immunoblot performed with indicated antibodies. *F*, H1395 cells were labeled and treated as indicated in schematic (*top*) followed by a DNA fiber assay. Each dot represents the ratio of CldU length to IdU length for an individual tract. Median is indicated. *p* value was calculated by Mann–Whitney test. n = 2, where at least 100 fibers were quantified for each biological replicate. *G*, H1395 cells were labeled and treated as indicated in schematic (*top*) followed by a DNA fiber assay. Each dot represents the ratio of CldU length to IdU length for an individual tract. Median is indicated. *p* value was calculated by Mann–Whitney test. n = 2, where at least 100 fibers were quantified for each biological replicate. *H*, HORMAD1 was depleted for 72 h in indicated LUAD cell lines. Cells were labeled and treated according to the schematic (*top*). Points represent the CldU/IdU ratio for individual tracts. Median is indicated. *p* value calculated by Mann–Whitney test; n = 3. *Below*: parallel lysates were obtained, and an immunoblot performed for indicated antibodies. 50kDA marker is indicated on each HORMAD1 blot. CldU, 5-chloro-2′-deoxyuridine; IdU, 5-iodo-2′-deoxyuridine;LUAD, lung adenocarincoma.

Depletion of HORMAD1 in LUAD cells increases sensitivity to DNA damaging agents, suggesting a function in the cellular response to genotoxic stress (40, 43, 44). To further elucidate the molecular mechanisms of HORMAD1 in tumor cells, we performed HORMAD1 immunoprecipitations followed by nano-HPLC mass spectrometry (MS) to identify endogenous HORMAD1 complexes in two LUAD cell lines, A549 and H1395, which express robust amounts of HORMAD1. This approach returned a cohort of proteins with annotated functions in DNA replication and mediators of replication stress response (Table S1, *A*–*C*). Specifically, HORMAD1 forms complexes with the DNA replication factors PCNA, RFC3, RFC4, ATAD5, and multiple mini-chromosome maintenance family members; as well as the replication stress response factor PARP1 and the fork protection factor, WRNIP1(Table S1, *A* and *C*) (46, 47, 48). Based on these findings, we evaluated whether HORMAD1 associates with replicating DNA using *in situ* analysis of protein interactions at DNA replication forks (SIRF) (49). We observed a robust association between HORMAD1 and 5-ethynyl-2′-deoxyuridine (EdU) in H1395 cells, which was diminished in the absence of EdU, HORMAD1 antibodies, and biotin antibodies, and cells where HORMAD1 is deleted (sgHORMAD1) (Figs. 1*A* and S1*A*). These data indicate that HORMAD1 is present at the sites of DNA replication.

Next, we aimed to investigate if HORMAD1 functions in DNA replication and/or the response to replication stress in cancer cells. For this analysis, we employed DNA fiber assays to monitor replication at the single molecule level (50). We deleted HORMAD1 using sgHORMAD1 in H1395 cells and verified depletion by immunoblotting (IB) (Fig. S1*A*). Importantly, we do not observe changes in cell cycle distribution or growth rates in H1395 sgHORMAD1 *versus* sgCTRL cells (Fig. S1, *B* and *C*). We evaluated the consequences of HORMAD1 loss on DNA replication in unperturbed conditions by sequentially pulse-labeling cells with the thymidine analogs 5-iodo-2′-deoxyuridine (IdU) and 5-chloro-2′-deoxyuridine (CldU). Comparison of the length of red and green tracks did not reveal any detectable differences in DNA replication between H1395 sgCTRL and sgHORMAD1 cells (median fiber length: sgCTRL= 16 μm, sgHORMAD1= 16 μm) (Fig. 1*B*). Next, we examined if HORMAD1 affected replication fork behavior after the induction of replication stress *via* treatment with hydroxyurea (HU). Specifically, the cells were pulse-labeled with IdU, then treated with HU, and finally pulse-labeled with CldU. DNA fiber analysis showed that exposure to HU induced a similar number of stalled forks in the H1395 sgCTRL and sgHORMAD1 cells (Fig. 1, *C* and *I*). Furthermore, H1395 sgCTRL and sgHORMAD1 cells exhibited a similar percentage of restarted forks (median sgCTRL: ∼71.86% *versus* sgHORMAD1: 74.32%) (Fig. 1*C*, ii), suggesting that HORMAD1 is not required for DNA replication fork restart following stalling. However, we observed a significant shortening of the IdU labeled tracts in H1395 sgHORMAD1 cells, suggesting that HORMAD1 is required for the integrity of the fork during replication stress (median sgCTRL: 12.39 *versus* sgHORMAD1: 9.40) (Fig. 1*C*, iii). To assess this possibility more directly, we sequentially labeled cells with IdU then CldU followed by HU exposure. In this scenario, the length of the CldU track reflects stability of newly synthesized DNA. In non-HU exposed samples, H1395 sgCTRL and sgHORMAD1 cells exhibited a similar ratio of CldU/IdU (median sgCTRL: 0.83 *versus* sgHORMAD1: 0.81) (Fig. 1*D*). Strikingly, after exposure to HU, we observed a substantial decrease in the CldU/IdU ratio in the H1395 sgHORMAD1 cells, reflecting a shortening of the CldU track (median sgCTRL: 0.82 *versus* sgHORMAD1: 0.52) (Fig. 1*D*). This result indicates that HORMAD1 promotes the protection of nascent DNA at stalled replication forks from degradation. Importantly, this defect is specific to HORMAD1, as ectopic expression of HORMAD1 was sufficient to rescue the decreased ratio (median sgHORMAD1: 0.57 *versus* HORMAD1-V5: 0.87) (Fig. 1*E*). We extended these findings to aphidicolin and camptothecin, both of which induce replication stress in H1395 cells. Exposure to these agents also required HORMAD1 to prevent nascent strand degradation (Fig. 1, *F* and *G*). Complete KO of HORMAD1 was not required for this phenotype, as siRNA-mediated depletion of HORMAD1 phenocopied the sgHORMAD1 defect (Fig. 1*H*, left panel). Furthermore, this phenotype is conserved in multiple genetic backgrounds as HORMAD1 depletion induced track shortening in four additional LUAD cell lines H650, H2023, H2126, and H358 (Fig. 1*H*).

### HORMAD1 protects the replication fork from nascent DNA degradation by MRE11-DNA2-Bloom

**Figure 2:**
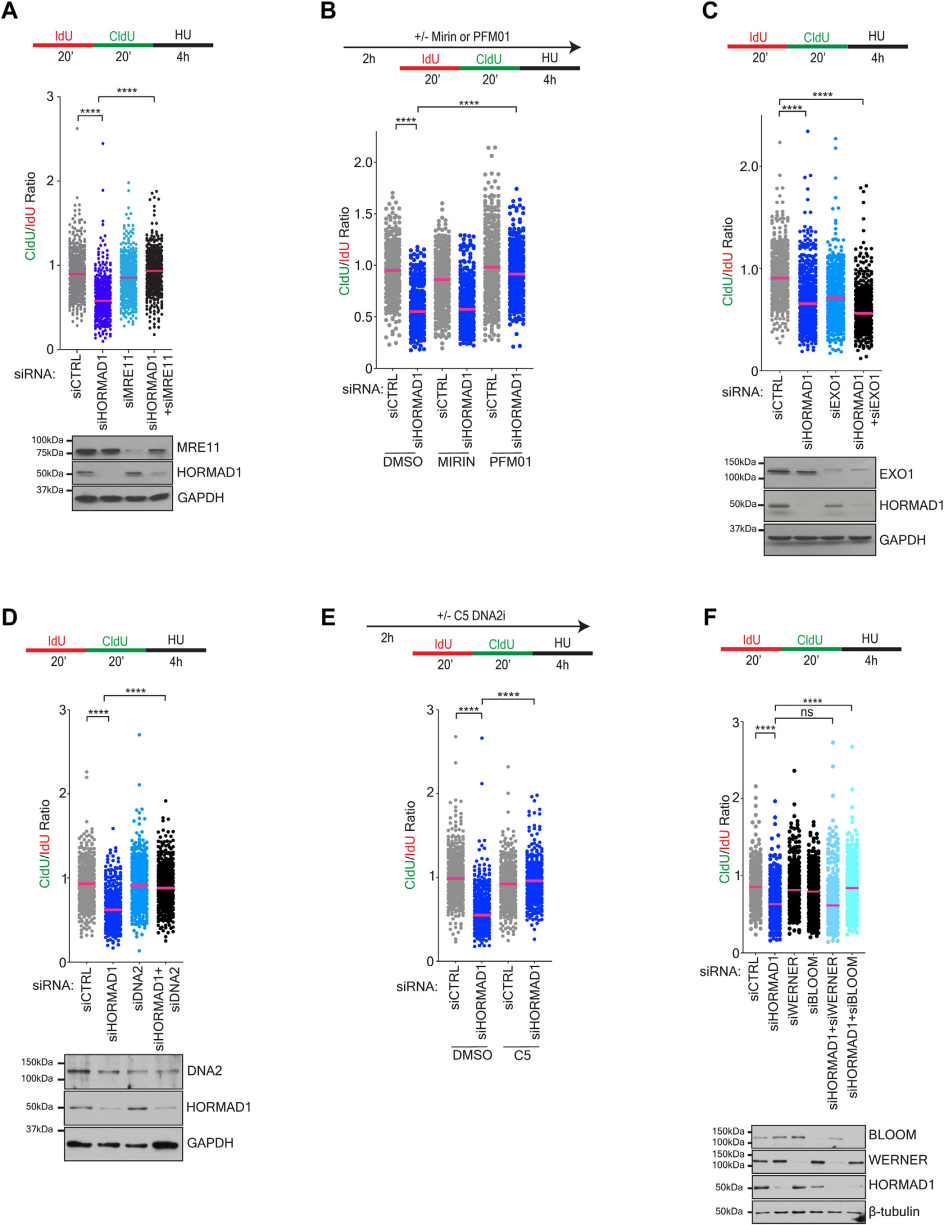
**HORMAD1 protects the replication fork from nascent DNA degradation by the MRE11-DNA2-BLM enzymatic axis**. *A*, H1395 cells were transfected with indicated siRNAs for 72 h before labeling according to the schematic (*top*) and a DNA fiber assay. Graph represents the CldU/IdU ratio for DNA fibers with the median indicated. *p* value calculated by Mann-Whitney *t* test; n = 3. *Below*: Parallel lysates were obtained and immunoblotted with indicated antibodies. *B*, H1395 cells were transfected with indicated siRNAs for 72 h followed by treatment according to the schematic (*top*). *p* value calculated by Mann–Whitney *t* test; n = 3. Graph as in *A*. *C* and *D*, as in *A*. *E*, H1395 cells were transfected with indicated siRNAs for 72 h. Cells were exposed to DMSO or C5 for 2 h prior to labeling (*top*) and DNA fiber assay. Graph represents the CldU/IdU ratio for individual fibers with median indicated. *p* value calculated by Mann–Whitney *t* test; n ≥ 3. *F*, as in *A*. CldU, 5-chloro-2′-deoxyuridine; DMSO, dimethylsulfoxide;IdU, 5-iodo-2′-deoxyuridine.

Degradation of stalled forks is typically catalyzed by unrestrained activity of nucleases, in particular MRE11 (48, 51). Indeed, depletion of MRE11 in combination with siHORMAD1 rescued the siHORMAD1 degradation phenotype in H1395 cells (siHORMAD1 median: 0.64 *versus* siHORMAD1+siMRE11 median: 0.98) (Fig. 2*A*). To determine if the endonuclease or exonuclease activity of MRE11 was responsible for the observed phenotype, we incubated siCTRL and siHORMAD1 cells with Mirin (exonuclease inhibitor) or PFM01 (endonuclease inhibitor) prior to IdU-CldU labeling and HU exposure to identify which MRE11 activity is mediating fork degradation. We observed that pretreatment with PFM01, but not Mirin, significantly rescued the fork degradation, which indicates that the endonuclease activity of MRE11 is the responsible activity (Fig. 2*B*).

MRE11 endonuclease activity often acts in concert with the long-range nucleases DNA2 and EXO1 (52). Thus, we codepleted HORMAD1 and either EXO1 or DNA2 and measured DNA degradation in H1395 cells (Fig. 2, *C* and *D*). This analysis revealed a rescue only upon the knockdown of DNA2. We observed comparable results using the DNA2 exonuclease inhibitor, C5 (Fig. 2*E*). DNA2 requires the Bloom or Werner helicase for unwinding DNA prior to digestion (53, 54). We depleted each helicase in combination with HORMAD1. In this setting, Bloom, but not Werner depletion, was observed to be sufficient to rescue the nascent DNA degradation in H1395 cells (Fig. 2*F*). Collectively, these findings indicate that HORMAD1 protects stalled replication forks from MRE11-DNA2-BLM nuclease attack.

### HORMAD1 promotes recruitment of RAD51 to stalled replication forks

**Figure 3:**
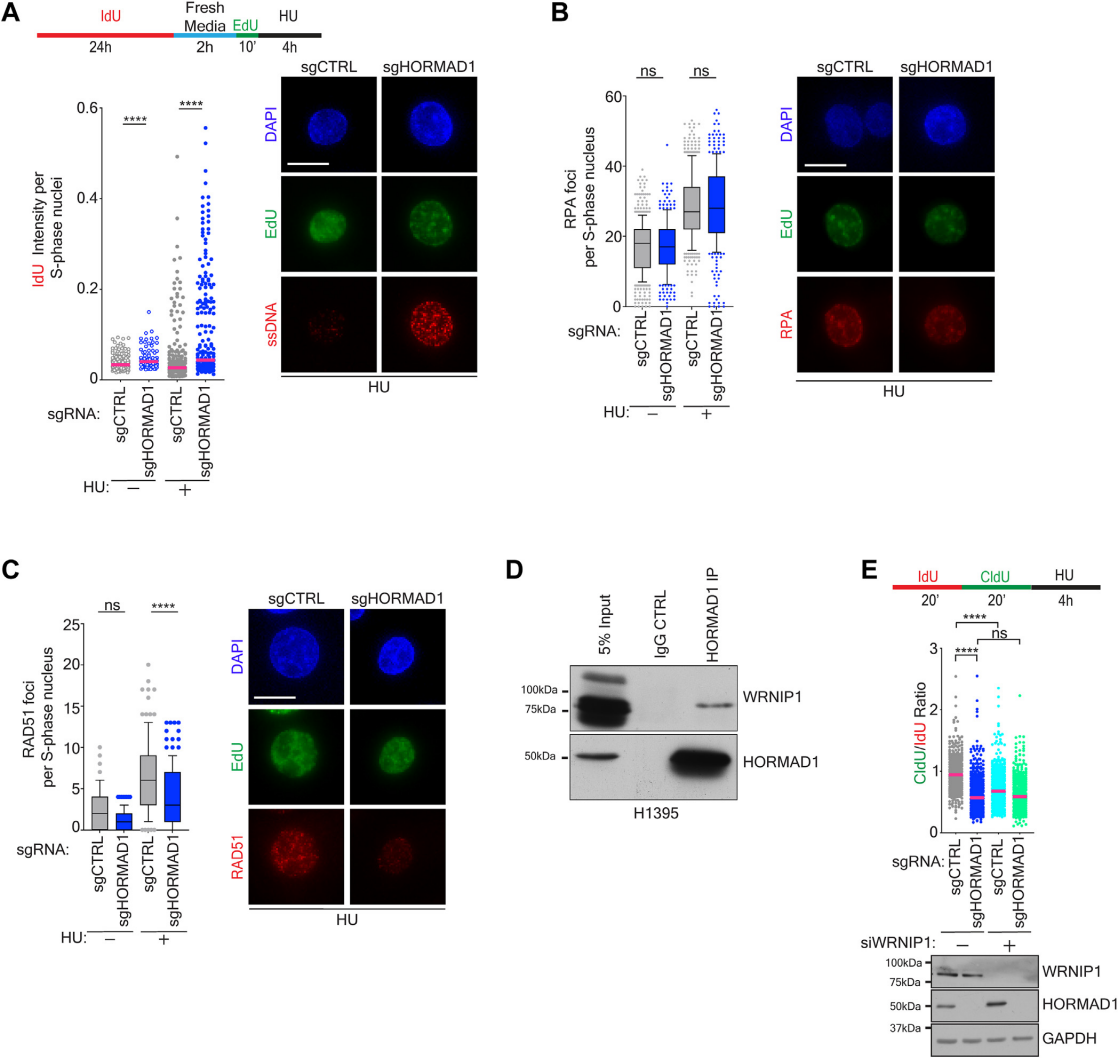
**HORMAD1 promotes recruitment of RAD51 to stalled replication forks**. *A*, H1395 cells were labeled as indicated in the diagram (*top*). Control cells were fixed following EdU labeling. Immunofluorescence was performed for IdU and EdU under nondenaturing conditions. Graph represents IdU nuclear fluorescence intensity in EdU positive cells. Mean is indicated. *p* value calculated by Mann–Whitney test; n = 3. *Right panel*: representative images of cells are shown. The scale bar represents 10 μm. *B*, indicated H1395 cells were labeled with EdU then exposed to HU or stained for RPA and EdU. Box plot was derived by measuring the mean RPA foci in EdU positive cells. *p* value calculated by Mann–Whitney test; n = 3. *Right panel*: representative images of cells are shown. The scale bar represents 10 μm. *C*, As in (*B*), except cells were stained for RAD51. *Right panel*: representative images of cells are shown. *D*, endogenous HORMAD1 was coimmunoprecipitated in H1395 cells and lysate samples were immunoblotted for HORMAD1 and WRNIP1. For loading, 5% of the input was taken from lysates and an IgG rabbit antibody pull-down was used as a control. Representative blot of n = 2. *E*, H1395 cells were transfected with indicated siRNAs for 72 h before labeling according to the schematic (*top*) and a DNA fiber assay. Graph represents the CldU/IdU ratio for DNA fibers with the median indicated. *p* value calculated by Mann–Whitney *t* test; n = 3. *Below*: Parallel lysates were obtained and immunoblotted with indicated antibodies. ICldU, 5-chloro-2′-deoxyuridine; dU, 5-iodo-2′-deoxyuridine; EdU, 5-ethynyl-2′-deoxyuridine; HU, hydroxyurea.

We next asked whether the nascent DNA degradation drives exposure of ssDNA. To assess this, we labeled parental DNA in cells by incubating them with IdU for 24 h followed by an EdU pulse to identify S phase cells, and then induced replication stress by addition of HU in H1395 cells. Under native conditions, Increased IdU signal would represent ssDNA resulting from nascent fork degradation during HU-induced replication stress. Notably, we observed a subtle, but significant increase in ssDNA exposure in non-HU treated sgHORMAD1 cells and this was significantly increased following HU treatment (Fig. 3*A*). Exposure of ssDNA leads to activation of the ataxia telangiectasia (ATR) checkpoint to arrest cell replication and mitigate damage. This checkpoint is intact in sgHORMAD1 cells as phosphorylation of ATR (p-Thr1989) and CHK1 (p-Ser345) were unchanged following HU treatment (Fig. S2*A*).

Exposed ssDNA is initially coated with Replication Protein A (RPA) and subsequently RAD51 to protect it from MRE11 mediated degradation. Thus, we examined the recruitment of these chromatin bound proteins in sgHORMAD1 compared to sgCTRL cells in the presence or absence of HU in S phase H1395 cells. We observed similar RPA focus formation in sgCTRL and sgHORMAD1 cells following HU exposure (Fig. 3*B*). However, we found a reduction in RAD51 recruitment in response to HU in sgHORMAD1 H1395 cells (Fig. 3*C*). Thus, our findings suggest that HORMAD1 is necessary for the recruitment of RAD51 to stalled replication forks. Based on these observations, we examined our MS analysis for interaction with fork protection factors (Table S1). We found that HORMAD1 associates with WRNIP1, which has previously been reported to recruit RAD51 to stall forks during replication stress (48). We confirmed that WRNIP1 and HORMAD1 form an endogenous complex in H1395 cells (Fig. 3*D*). We next asked whether codepletion of WRNIP1 alters DNA degradation following loss of HORMAD1 upon replication stress. As previously described, WRNIP1 depletion (siWRNIP1) led to degradation of stalled forks (48). Depleting WRNIP1 in sgHORMAD1 cells did not substantially change the degree of degradation compared to sgHORMAD1 alone (median sgHORMAD1: 0.57 *versus* siWRNIP1 + sgHORMAD1: 0.59) (Fig. 3*E*). As combined depletion did not exacerbate the phenotype, we conclude that the extent of degradation of siHORMAD1 was epistatic to that of siWRNIP1 (Fig. 3*E*). This finding suggests that HORMAD1 and WRNIP1 may operate in a similar pathway with respect to fork protection.

### HORMAD1 protects against DNA and chromosomal damage

**Figure 4:**
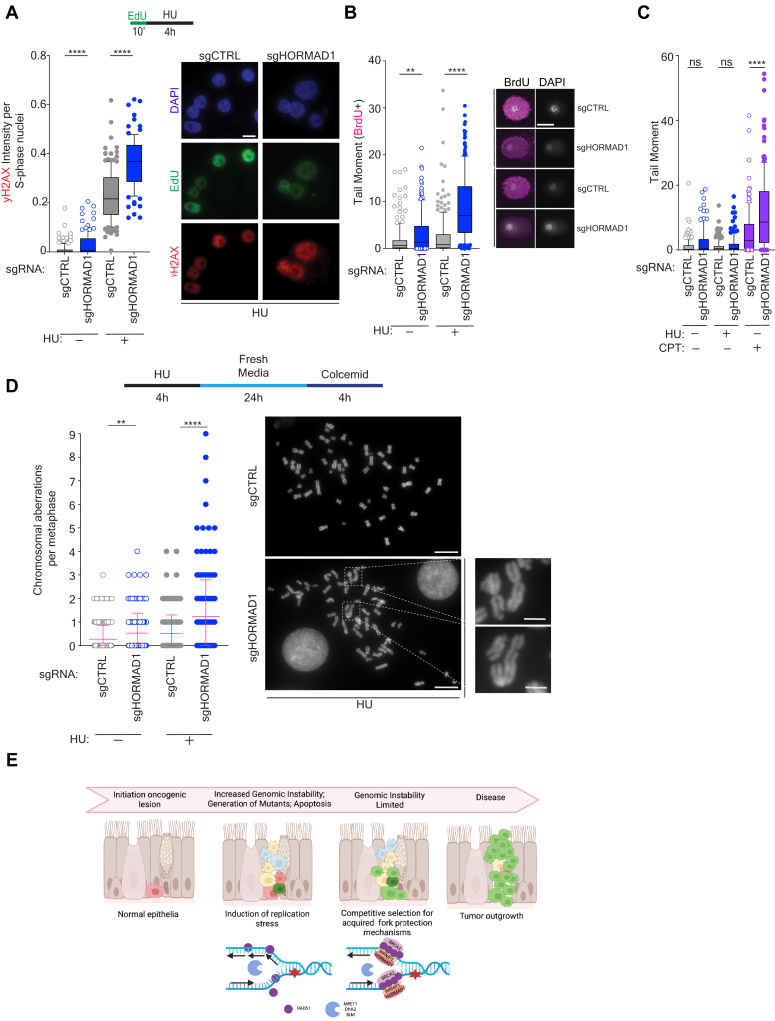
**HORMAD1 mitigates DNA damage during DNA replication stress**. *A*, indicated H1395 cells were labeled with EdU for 10 min then exposed to HU or fixed. Immunofluorescence was performed for mean γH2AX and EdU. *Center line* indicates the median, bounds of box indicate the first and third quartile, and whiskers indicate the 10th and 90th percentile for mean γH2AX nuclear fluorescence intensity in EdU positive cells. The scale bar represents 10 μm; *p* value calculated by Mann–Whitney test; n ≥ 3. *Right panel*: representative images of cells are shown. *B*, indicated H1395 cells were labeled with BrdU for 20 min and processed immediately or exposed to HU and then processed for an alkaline comet assay. *Left*: Box plot was derived from measuring the tail moment in BrdU positive cells and boundaries are indicated as in (*A*). *p* value calculated by Mann-Whitney test; n = 3. *Right*: representative images of comets are shown. The scale bar represents 50 μm. *C*, indicated H1395 cells were processed immediately or exposed to either HU or 1 μm Camptothecin and processed for a neutral comet assay. *Left*: Box plot was derived from measuring the tail moment in all cells and boundaries are indicated as in (*A*). *p* value calculated by Mann–Whitney test; n = 2. *D*, H1395 cells were treated as indicated in schematic (*top*) prior to performing a metaphase spread assay. Each data point represents one metaphase spread. Mean is indicated. Error bars represent ± SEM. *Right*: Representative pictures magnified from 100×. The scale bar represents 10 μm. Inset scale bar represents 2.5 μm. *p* value calculated by Mann-Whitney test; n = 3 experiments, with a minimum of 30 metaphases per condition per experiment. *E*, schematic indicating tumor evolution and replication stress mitigation by HORMAD1. Created with BioRender.com. BrdU, bromodeoxyuridine; EdU, 5-ethynyl-2′-deoxyuridine; HU, hydroxyurea.

Given our findings that HORMAD1 promotes ssDNA protection, we hypothesized that loss of HORMAD1 could increase genomic instability following replication stress. To evaluate this possibility, we first measured γH2AX signal (an indirect marker for DSBs) in cells lacking HORMAD1. We observed an increase in signal in sgHORMAD1 cells than sgCTRL at baseline, suggesting HORMAD1 protects tumor cells from endogenous DNA damage (Fig. 4*A*). In response to HU, the γH2AX signal was markedly increased in the sgHORMAD1 cells than sgCTRL (Fig. 4*A*). Using an alkaline comet assay, which can detect multiple types of DNA damage including ssDNA and DSB breaks, we observed a significant increase in tail moment following exposure to HU in sgHORMAD1 cells (Fig. 4*B*). Importantly, we did not observe an increase in DSBs in sgHORMAD1 cells as compared to sgCTRL cells at baseline or in response to HU, despite observing a robust increase following high dose (1 μM) camptothecin exposure (Fig. 4*C*). This control indicates that HU treatment did not induce DBSs, and the resection we observe in the absence of HORMAD1 likely stems from unprotected DNA replication forks. To evaluate the consequences of this observed DNA damage on chromosomal integrity, we performed metaphase spreads. As expected of cancer cells, H1395 sgCTRL cells displayed a low but detectable incidence of chromosomal aberrations including fragmented chromosomes and chromatid breaks following HU treatment. In H1395 sgHORMAD1 cells, accumulation of chromosomal aberrations was significantly increased in untreated samples and dramatically induced upon HU exposure (Fig. 4*D*). This finding suggests that HORMAD1 is essential for chromosomal integrity in LUAD cells.

## Discussion

Meiotic proteins are often aberrantly expressed in tumor cells. If and how these abnormally expressed proteins are engaged in DNA metabolism has only recently begun to be addressed (55). HORMAD1 is the best studied of these meiotic cancer genes and it is well established that HORMAD1 expression correlates with an increase in mutation burden, neo-antigen production, and gross chromosomal abnormalities in cancer (39, 40, 43, 44). One major question that has emerged is whether HORMAD1 is a cause or consequence of increased DNA damage. Two reports have suggested HORMAD1 promotes genomic instability when inappropriately expressed by biasing cancer cells toward using the error-prone DNA repair pathways translesion synthesis, nonhomologous end joining, or by blocking DNA mismatch repair (39, 41, 42). Alternatively, evidence suggests that HORMAD1 can promote repair of DSBs *via* the error-free pathway HR, suggesting it also contributes to genome integrity (40, 43, 44). Our study adds more evidence to the possibility that HORMAD1 can mitigate excessive genomic instability by reducing the accumulation of genomic damage during replication stress. This activity would permit continued rounds of proliferation and viability of tumor cells without accumulation of excessive DNA damage.

During DNA replication, ssDNA is exposed in short stretches between Okazaki fragments and in stretches on the leading strand due to uncoupling of helicase and polymerase processivity (56). This process is exacerbated by replication stress, which is rampant in cancer cells due to unscheduled origin firing, depletion of dNTP pools, or generation of R-loops. The chronic exposure of ssDNA increases the risk of DSBs, fork collapse, chromosome fusions, and ultimately the loss of genetic information (57). Thus, cancer cells are in a precarious position in which they must maintain proliferation, but failure to protect excessive ssDNA could lead to catastrophic genomic instability. Indeed, the chronic state of replication stress in tumors has been implicated in limiting available pools of the ssDNA protection factor, RPA (58). While low levels of DNA damage could generate mutants that allow for adaptation, the effectiveness of DNA-damage chemotherapeutics suggests that cancer cells cannot tolerate excessive damage. Thus, we postulate that HORMAD1 is engaged to promote protection of ssDNA during replication and replication stress in LUAD. This protection appears to be due to a HORMAD1-dependent localization of RAD51 to ssDNA. Importantly, previous studies have implicated HORMAD1 in recruitment of RAD51 to γ-irradiation-induced DSBs, suggesting a conserved mechanism in supporting RAD51 nucleofilament formation irrespective of the damage (44). In developing gametes, HORMAD1 associates with DNA and serves as a platform for a multiprotein complex to recruit SPO11(28). In tumors, HORMAD1 could be acting in a similar manner to promote fork protection through its association with the AAA+ ATPase, WRNIP1, and the recruitment of fork protection factors including RAD51(49). Importantly, HORMAD1 interacts with additional AAA+ ATPases in sperm, thus suggesting a potential conserved mode of interaction (59). Alternatively, it is possible that HORMAD1 alters the required stoichiometry of the factors required for DNA fork protection, preventing exhaustion of these proteins that protect ssDNA (58). While the mechanism of how HORMAD1 recruits RAD51 to chromatin remains unknown, our studies an additional phenotypic system to further evaluate the biochemical nature of HORMAD1’s relationship to RAD51 recruitment in future studies (40, 44).

Our studies have focused on HORMAD1’s function in transformed cells. Notably, a recent study found that HORMAD1 expression is highly upregulated in nonmalignant cells that are able to overcome a proliferation block due to cyclin E overexpression (60). Thus, it is possible that HORMAD1 is engaged early on during transformation in premalignant cells where excessive replication stress due to oncogene activation reduces cell growth and serves as a barrier to transformation (61).

The multiple studies of HORMAD1 function in tumors suggest a mixture of genomic protection and disruption, depending on the stage and type of cancer or additional changes in DNA repair pathways (BRCA1/2 mutation, ATM/ATR loss, and so on). This apparent flexibility in HORMAD1 function could be due to its capacity to bind to chromatin as well as a variety of replication and repair proteins and serve as a platform for protein complex formation (Table S1, *A*–*C*, (39, 42)). Despite the disparate findings in these studies, one theme is strikingly consistent: loss of HORMAD1 increases the sensitivity of tumors to DNA damage agents including ionizing radiation, replication stress, PARP inhibitors or 6-thioguanine treatment, suggesting that cotargeting HORMAD1 could represent a mechanism to dramatically increase the log-kill of these anticancer agents without compromising their narrow therapeutic window (39, 40, 43, 44).

## Experimental procedures

### Cell lines and culture conditions

All NSCLC cell lines were obtained from John Minna (UT Southwestern) between 2014 and 2018. U2OS cells were obtained from Michael White (UT Southwestern). NSCLC cell lines were maintained in Roswell Park Memorial Institute medium (RPMI-1640; Sigma Aldrich) supplemented with 5% fetal bovine serum (Sigma-Aldrich) and incubated at 37 °C in a humidified 5% CO_2_ atmosphere. HEK293T cells were obtained from *American Type Culture Collection* in 2014 and cultured in Dulbecco's modified Eagle's medium supplemented with 10% fetal bovine serum at 37 °C in a humidified 5% CO_2_ atmosphere. All cells were periodically evaluated for *mycoplasma* contamination by a *mycoplasma* PCR detection kit (Cat# G238, ABM). Authenticity of cell lines was evaluated by detection of ten genetic loci using the GenePrint 10 System (Promega) and cross referencing to *American Type Culture Collection* or internal genetic profiles.

### Inhibitors

HU (Chem-Impex International (50–525–181)) was used at a final concentration of 4 mM for 4 h. The MRE11 Exonuclease inhibitor, Mirin, (Cayman Chemicals (13208)) was used at a final concentration of 50 μM. The MRE11 Endonuclease inhibitor, PFM01 (Sigma-Aldrich (SML1735-5MG)) was used at a final a concentration of 10 μM. The DNA2 C5 inhibitor (AOBIOUS (NSC15765)) was used at a final concentration of 20 μM. Aphidicolin (Cayman Chemicals (14007)) was used at a final concentration of 2 μM. Camptothecin (Cayman Chemicals (11694)) was used at a final concentration of 100 nM.

### Antibodies

Antibodies used: γH2AX (05–636, EMD Millipore) (1:1000), RPA2 (ab2175, Abcam) (1:1000), RAD51 Immunofluorescence (ABE257, Sigma-Aldrich) (1:100), RAD51 Immunoblot (D4B10, CST) (1:1000), HORMAD1 (HPA037850, Sigma-Aldrich) (1:5000), CHK1 (2360S, CST) (1:1000), pCHK1 (S345) (2348S,CST) (1:1000), ATR (2790S, CST) (1:1000), pATR (T1989) (58014S,CST) (1:1000), CldU (ab6326, abcam) (1:500), IdU (347,580, BD Biosciences) (IF 1:50, DNA fiber assay 1:500), bromodeoxyuridine (BrdU) (5292S, CST) (1:100), Vinculin (sc-73614, Santa Cruz) (1:3000), GAPDH (G8795, Sigma-Aldrich) (1:10,000), MRE11 (NB100–142, Novus Biologicals) (1:1000), EXO1 (A302–640A, Bethyl Labs) (1:1000), DNA2 (ab96488, Abcam) (1:1000), BLM (A300–110A-M, Bethyl Labs) (1:1000), Beta-Tubulin (2128S, CST) (1:5000), WRNIP1 (NB110–61626, Novus Biologicals), Normal Rabbit IgG (2729S, CST), Ku80 was described previously (62).

### Transfections

For siRNA transfections, cells were trypsinized and seeded in Opti-MEM containing Lipofectamine RNAiMAX (Thermo Fisher Scientific) complexed with siRNAs (final concentration 50 nM). siRNAs were purchased from Sigma-Aldrich as follows: nontargeting controls (VC30002), targeting HORMAD1 (SASI_Hs01_00222714, SASI_Hs01_00222715, SASI_Hs01_00222716), MRE11 (SASI_Hs02_00339885), EXO1 (SASI_Hs01_00076269), DNA2 (SASI_Hs02_00314950), WRN (SASI_Hs01_00219502), BLM (SASI_Hs01_00103851, SASI_Hs01_00103853),WRNIP1 (SASI_Hs01_00169895, SASI_Hs01_00169893, SASI_Hs01_00169894, SASI_Hs01_00169896). If more than one siRNA is indicated, these were combined into a pool (total siRNA final concentration of 50 nM) and used for the respective experiment.

### Lentiviral transduction

Stable cell lines were generated through lentiviral-mediated transduction. HEK293T cells were cotransfected with the target gene vectors (in pLX304) and lentiviral packaging plasmids (psPAX2 and pMD2.G). Forty-eight hours later, virus-conditioned medium was harvested, passed through 0.45 μm pore-size filters, and then used to infect target cells in the presence of Sequa-brene (S2667, MilliporeSigma) for at least 6 h. Six hours later, medium was replaced, and cells were allowed to recover. Stable sgCTRL and sgHORMAD1 cell lines were generated using pLX-sgRNA and pCW-Cas9 constructs (Addgene plasmid #50662, #50661) as described previously (44). H1395 cells were infected with pCW-Cas9 and selected with puromycin for 3 days. Next, pCW-Cas9 stable cells were then infected with pLX-sgHORMAD1, selected with blasticidin, and treated daily with 1 μg/ml doxycycline for 2 weeks to induce Cas9 expression and HORMAD1 deletion. Immunoblotting was used to check for full HORMAD1 KO. Sequence for PLX-sgHORMAD1 (TCCCCTCAAATACGTGATA), PLX-sgGFP (GGGCGAGGAGCTGTTCACCG).

### Endogenous coimmunoprecipitation and liquid chromatography-tandem mass spectrometry based HORMAD1 interactome analysis

Cells were lysed for 30 min on ice in nondenaturing lysis buffer (NDLB) (50 mM Hepes pH 7.4, 150 mM NaCl, 1.0 % Triton X-100, 0.5% sodium deoxycholate, 25 mM β-glycerophosphate, 1 mM EDTA, 1 mM EGTA, 1 mM Na_3_VO_4_, 1 μg/ml pepstatin, 2 μg/ml leupeptin, 2 μg/ml aprotinin, and 10 μM bestatin). Lysates were clarified at 12,000*g* for 10 min and precleared with Protein A/G agarose beads for 1 h at 4 °C. Five percent of each lysate was set aside as input material and the remainder was immunoprecipitated with appropriate antibodies (1.5 μg of antibody) overnight at 4 °C. Protein A/G agarose beads blocked with 0.5% bovine serum albumin (BSA) overnight. Beads were then washed three times in NDLB and 50 μl of beads incubated with lysate for at least 2 h. Beads were then washed in NDLB three times for 5 min and eluted in hot 2× sample buffer. Immunoprecipitated proteins were separated by SDS-PAGE and stained with Colloidal Coomassie blue. Bands were excised from the acrylamide gel and in-gel tryptic digestion was performed. For MS, samples were digested overnight with trypsin (Pierce) following reduction and alkylation with DTT and iodoacetamide (Sigma–Aldrich). The samples then underwent solid-phase extraction cleanup with an Oasis HLB plate (Waters) and the resulting samples were injected onto an Orbitrap Fusion Lumos MS coupled to an Ultimate 3000 RSLC-Nano liquid chromatography system. Samples were injected onto a 75 um i.d., 75-cm long EasySpray column (Thermo Fisher Scientific) and eluted with a gradient from 0 to 28% buffer B over 90 min. Buffer A contained 2% (v/v) acetonitrile and 0.1% formic acid in water, and buffer B contained 80% (v/v) acetonitrile, 10% (v/v) trifluoroethanol, and 0.1% formic acid in water. The MS operated in positive ion mode with a source voltage of 1.5 kV and an ion transfer tube temperature of 275 °C. MS scans were acquired at 1,200,00× resolution in the Orbitrap and up to 10 MS/MS spectra were obtained in the ion trap for each full spectrum acquired using higher-energy collisional dissociation for ions with charges 2 to 7. Dynamic exclusion was set for 25 s after an ion was selected for fragmentation.

Raw MS data files were analyzed using Proteome Discoverer v2.4 SP1 (Thermo Fisher Scientific; https://www.thermofisher.com/us/en/home/industrial/mass-spectrometry/liquid-chromatography-mass-spectrometry-lc-ms/lc-ms-software/multi-omics-data-analysis/proteome-discoverer-software.html), with peptide identification performed using Sequest HT searching against the human protein database from UniProt. Fragment and precursor tolerances of 10 ppm and 0.6 Da were specified, and three missed cleavages were allowed. Carbamidomethylation of Cys was set as a fixed modification, with oxidation of Met set as a variable modification. The false-discovery rate cutoff was 1% for all peptides. Label-free quantitation of proteins across samples was performed using SINQ normalized spectral index Software (63; https://analyticalsciencejournals.onlinelibrary.wiley.com/doi/10.1002/pmic.201000800).

### *In situ* analysis of protein interactions at DNA replication forks (SIRF) assay

Cells were plated on coverslips for 24 h before incubating with 20 μM EdU for 10 min. Cells were then fixed with 3.7% paraformaldehyde in PBS (pH 7.4) for 15 min at room temperature (RT) and permeabilized with 0.25% Triton X-100 in PBS for 10 min at RT. Cells were washed with PBS twice for 5 min each. The click reaction cocktail (2 mM CuSO4 (copper sulfate), 20 μM biotin azide, and 100 mM sodium ascorbate in PBS) was added to each coverslip and cells were incubated at RT for 30 min. Cells were then blocked in blocking buffer from the manufacturer’s kit for 1 h at RT. Cells were incubated with primary antibodies overnight at 4 °C (mouse anti-biotin antibody (1:250, B7653, Sigma-Aldrich) with rabbit anti-HORMAD1 antibody (HPA037850, Sigma-Aldrich)). Cells were washed twice with PBS and incubated with premixed Duolink PLA plus and minus probes for 1 h at 37  °C. The subsequent steps in proximal ligation assay were carried out using the Duolink PLA Fluorescence Kit (Sigma-Aldrich) according to the manufacturer’s instructions. Slides were stained with 4′,6-diamidino-2-phenylindole (DAPI) and cells were then imaged using a Keyence BZ-X700 microscope at 100× magnification. SIRF assays without biotin antibody (target protein antibody only) or EdU incubation or in sgHORMAD1 cells were performed as negative controls to confirm the specificity of the SIRF signal.

### DNA fiber assays

Cells were pulse-labeled with 20 μM IdU then 200 μM CldU as indicated in the experimental schemes. For immunodetection of labeled tracks, the following primary antibodies were used: anti-CldU (rat monoclonal anti-BrdU/CldU; BU1/75 ICR1 Abcam, 1:500) and anti-IdU (mouse monoclonal anti-BrdU/IdU; clone b44 Becton Dickinson, 1:500). The secondary antibodies used were goat anti-rat Alexa Fluor 488 or goat anti-mouse Alexa Fluor 596 (Thermo Fisher Scientific, 1:1000). The incubation with antibodies was accomplished in a humidified chamber for 1 h at RT. ProLong Gold Antifade reagent with DAPI was used to mount slips on glass slides and images were acquired by a Leica DM5500 B upright microscope at a 60× magnification. Length of labeled tracks was measured using Image J (https://ij.imjoy.io), and a minimum of 100 individual fibers were analyzed per biological replicate. At least two biological replicates were performed.

### Immunofluorescence assays

Immunoflourescence assays were used to visualize γH2AX, RAD51, RPA, and parental ssDNA. Where specified, EdU (Cayman chemicals (#20518)) was used at final concentration of 20 μM for 10 min. Cells were plated on coverslips 24 h before commencing the experiment to allow for attachment. The cells were treated with PBS or 4 mM of HU for 4 h. For nuclear preextraction cells were washed with PBS and cytoskeletal buffer (CSK) (10 mM Pipes, pH 6.8, 100 mM NaCl, 300 mM sucrose, 3 mM MgCl_2_, ± 0.3% Triton X-100) once. Cells were then incubated for 5 min with Triton-CSK buffer (0.3% Triton-X) and washed with CSK buffer (no Triton) four times. Cells were washed in cold PBS, followed by fixation with cold 3.7% formaldehyde. Cells were then washed twice with PBS and incubated in 0.25% Triton for 10 min prior to washing three times with 1× PBS. Next coverslips were blocked for 30 min at RT in blocking buffer (1% BSA in 0.001% Tris buffered saline (20 mM Tris, 150 mM NaCl, 0.1% Tween-20) (TBST) mixed with PBS). Primary antibodies were diluted with blocking buffer and incubated with samples overnight at 4 °C. Next day, samples were washed three times in PBS for 5 min each, followed by incubation with Alexa Fluor–conjugated secondary antibodies (Thermo Fisher Scientific) at a dilution of 1:1000 for 1 h at RT. Finally, samples were washed three times for 5 min with PBS at RT, followed by mounting onto glass slides using ProLong Gold Antifade reagent with DAPI. Images were acquired by epifluorescence microscope Keyence BZ-X700.

For parental ssDNA detection, cells were seeded on coverslips in a 24-well plate for 24 h. Subsequently, cells were pulsed for 24 h with IdU (20 μM), washed twice with PBS, incubated in fresh media for 2 h, and subsequently either incubated with PBS or 4 mM HU for 4 h. At the time of collection, samples were preextracted and processed as described above for immunofluorescence in native conditions. Cells were then imaged using a Keyence BZ-X700 microscope at 60× magnification. Mean fluorescence intensity or foci count was determined using CellProfiler Software (https://cellprofiler.org).

### Cell lysis and IB

Samples were lysed in preheated (100 °C for 5 min) 2× Laemmli sample buffer with Beta-mercaptoethanol and boiled for 5 min. Samples were resolved using SDS-PAGE, and transferred to an Immobilon polyvinylidene fluoride membrane (MilliporeSigma), blocked in 5% nonfat dry milk followed by incubation in the respective primary antibodies overnight. Following incubation, membranes were washed three times with TBST, and incubated for 1 h with horseradish peroxidase-coupled secondary antibodies (Jackson Immunoresearch). Subsequently, membranes were washed three times with TBST and then developed using SuperSignal West Pico PLUS chemiluminescence substrate (Thermo Fisher scientific, 45–000–875). Immunoblots were scanned using the EPSON perfection v700 photo scanner.

### Cell cycle analysis and cell number analysis

Two million cells were seeded on a 10-cm^3^ dish 24 h prior to the onset of the experiment. Cells were washed once with PBS followed by trypsinization then cell pellets obtained were washed again with PBS. Cells were then fixed in ice-cold 70% EtOH at −20 °C for 30 min. Next, cells were washed with PBS and resuspended in DNA extraction buffer (0.2 M Na_2_HPO_4_, 0.1 M citric Acid: pH 7.8) at for 20 RT min. Cells were subsequently centrifuged, supernatant was removed, and cells were stained with propidium iodide solution (Thermo Fisher Scientific (AAJ66584AB), 80 μg/ml; 0.1% Triton X-100; 100 μg/ml RNase A in PBS) in the dark for 20 min. Cells were immediately analyzed by flow cytometry using a BD LSR Fortessa instrument and BD FACSDiva 6.2 software (https://www.bdbiosciences.com/en-nl/products/software/instrument-software/bd-facsdiva-software). A minimum of 5000 cells were analyzed per condition. FlowJo software (https://www.bdbiosciences.com/en-nl/products/software/flowjo-v10-software) was used to generate flow charts and calculate KS-Max difference.

For cell proliferation assays, cells were seeded at a density of 50,000 cells per well on a 6-well plate. Each day for 6 days a single well was trypsinized and counted to measure the number of cells. At least three biological replicates were performed. Cells were counted using Bio-rad TC-10 automated cell counter, and results were graphed using Graphpad prism (https://www.graphpad.com).

### Subcellular fractionation

Subcellular fractionations were performed using the Thermo Fisher Scientific subcellular fractionation kit (Cat#78840). Protein content was measured using the Pierce BCA protein assay kit (Cat#23225). Followed by SDS-PAGE and IB. Blots were scanned on an EPSON perfection v700 photo scanner. Band intensity was measured using Image J for each respective protein band. Normalization was done first for loading using Ku80. p-ATR and p-CHK1 were normalized to their total unphosphorylated counterparts following normalization to total loading.

### Alkaline and neutral comet assay

A total of 250K cells were seeded on 6-well plates for 24 h. BrdU (Cayman chemicals (#15580)) was used at a final concentration of 20 μM for 20 min. Cells were incubated in 4 mM HU for 4 h prior to cell collection and comet assay in accordance with the manufacturer's instructions (R&D #4250–050-K). Briefly, cells were suspended in cold 1 ml of cold PBS and diluted 1:10 with low melting agarose to achieve a final concentration of ∼1000 cells per well on a comet slide. The slide was then incubated at 4 °C for 30 min. Slides were lysed overnight at 4 °C with provided lysis buffer. Slides were incubated in alkaline unwinding solution or in neutral solution at RT for 20 min (pH >12). Slides were then electrophoresed at 25 V for 30 min at 4 °C in the dark. Alkaline comet Slides were incubated in H_2_O twice for 5 min and 70% EtOH once. For neutral comets, slides were immersed in DNA precipitation solution for 30 min RT followed by incubation in 70% EtOH for 30 min RT. Neutral Comet slides were air dried for 15 min at 37 °C. Neutral comet slides were then stained with SYBR Gold (S11494, Thermo Fisher Scientific) for 30 min RT, and then air dried for 15 min at 37 °C. For alkaline comet assay, slides were dried for 15 min at 37 °C and then washed with two changes of PBS for 5 min each and blocked with blocking buffer (PBS containing 0.1% BSA and 0.1% Tween 20), for 30 min at RT. The slides were then incubated with mouse monoclonal anti-BrdU (1∶100) diluted in blocking buffer in the dark in a humidified box at RT for 1 h. The excess of primary antibody was washed off with three changes of PBS and once with blocking buffer and probed with secondary antibody (Alexa Fluor 488–labeled goat anti-mouse antibody (1∶1000) diluted in blocking buffer for 1 h. Following incubation, cells were washed as previously mentioned, air dried for 15 min at 37 °C, then mounted onto glass slides using ProLong Gold Antifade reagent with DAPI. Images were acquired by epifluorescence microscope Keyence BZ-X700 at a 10× magnification. Tail moments were scored using the CometScore software (TriTek; http://rexhoover.com/index.php?id=cometscore).

### Metaphase spread

Two million cells were seeded in a 10 cm^3^ dish. Twenty-four hours later, samples were treated with 4 mM HU or vehicle (PBS) for 4 h, followed by a PBS wash and incubation in fresh media for 24 h. Subsequently, cells were treated with 0.2 μg/ml Colcemid for 4 h (KaryoMAX). Mitotic shake-off was carried out and the cells collected from the media were incubated in prewarmed (37 °C) 75 mM KCl for 15 min. Cells were then resuspended in prechilled Carnoy fixative (3:1 methanol:acetic acid), pelleted, and resuspended in cold Carnoys fixative. The samples were placed in a dropwise manner onto slides, allowed to air dry, and mounted in ProLong Gold Antifade reagent with DAPI. Metaphase spreads were then imaged using a Keyence BZ-X700 microscope at 100× magnification. Chromosomal aberrations of fragments, individual chromatids, dicentric, breaks were quantitated manually. A minimum of 30 spreads were evaluated per condition, per experiment for a minimum of 120 spreads for each condition in three independent experiments.

### Statistical analysis

Graphpad Prism (Graphpad Software) was used to perform statistical analyses. Normality of data was determined by the Shapiro–Wilk normality test. Outliers were removed by the ROUT outlier identification method in Graphpad Prism. For Box and Whisker graphs, the center line indicates the median, bounds of box indicate the first and third quartile, and whiskers indicate the 10th and 90th percentile. Statistical differences in all cases were determined by Mann–Whitney or *t* test as specified in the figure legends.: ns= not significant, *p* > 0.05; ∗*p* < 0.05; ∗∗*p* < 0.01; ∗∗∗*p* < 0.001; ∗∗∗∗*p* < 0.0001.

## Data availability

Mass spectrometry data were deposited into the MassIVE database under accession: MSV000091966. All other data is contained within this manuscript.

## Supporting information

This article contains supporting information.

## Conflict of interest

The authors declare that they have no conflict of interest with the contents of this article.
