# Supplementary figures and images for: The cancer testes antigen, HORMAD1, limits genomic instability in cancer cells by protecting stalled replication forks
Source: J Biol Chem. 2023 Oct 12;299(11):105348. doi: 10.1016/j.jbc.2023.105348 (PMC10656231; doi:10.1016/j.jbc.2023.105348)

Supplemental Figure 1

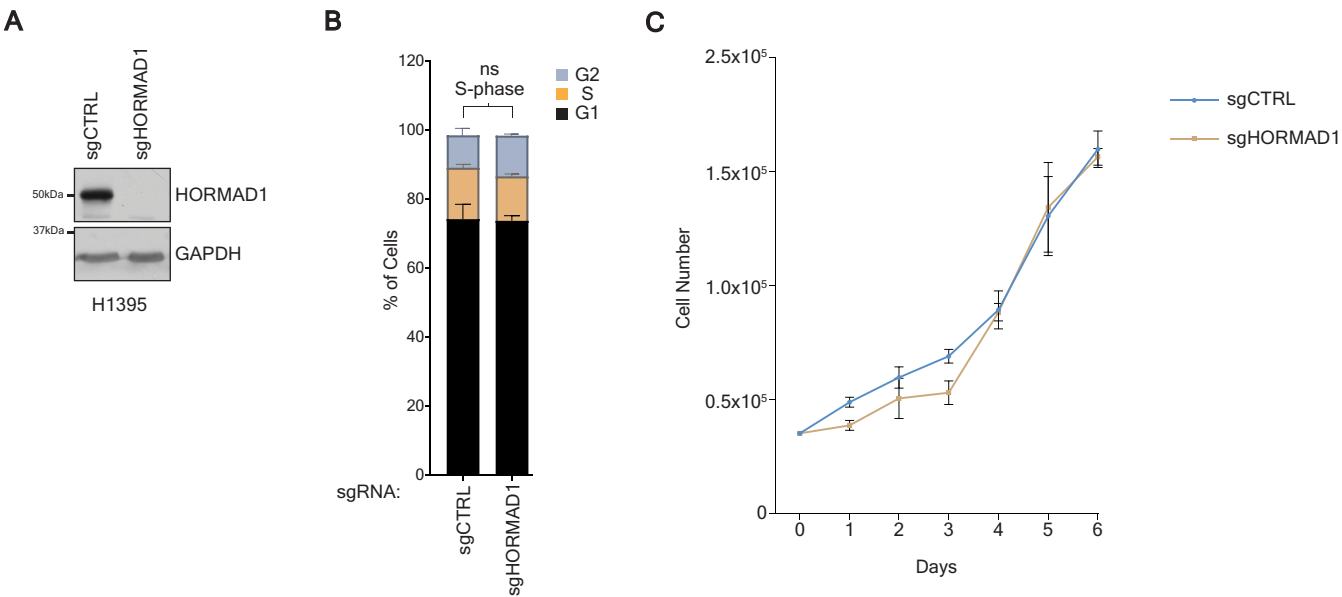

Supplemental Figure 2

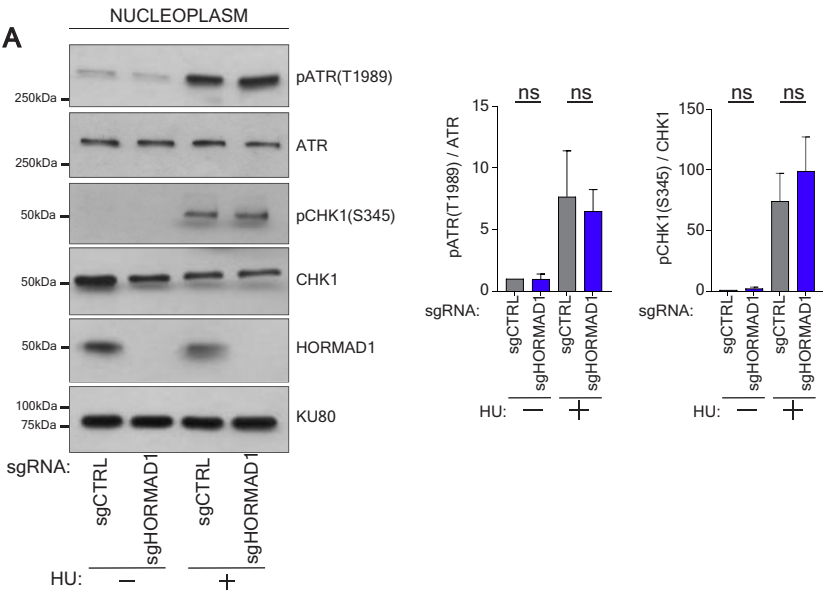

Supplement: Supplemental data [file mmc1.pdf]
