# Supplementary material for: The cancer testes antigen, HORMAD1, limits genomic instability in cancer cells by protecting stalled replication forks
Source: J Biol Chem. 2023 Oct 12;299(11):105348. doi: 10.1016/j.jbc.2023.105348 (PMC10656231; doi:10.1016/j.jbc.2023.105348)
Supplement: Supplemental legends [file mmc3.pdf]

Supplemental Table 1A. List of HORMAD1 interacting partners involved in DNA metabolism in A549 and H1395 cells.

Supplemental Table 1B. List of all HORMAD1 interacting partners from A549 cells.

Supplemental Table 1C. List of all HORMAD1 interacting partners from H1395 cells.

Supplemental Figure 1. HORMAD1 knockout in H1395 lung adenocarcinoma cells does not affect cell cycle profile or cell proliferation.

(A) Indicated H1395 cells were processed for cell cycle stage based in PI staining. Error bars represent SEM, n=3. P value calculated by an unpaired t-test. **(B)** Indicated H1395 cells were seeded at density of 50k cells and counted each day for six days. Each point represents the mean cell number ± SEM for n=3. P value calculated by an unpaired t-test.

Supplemental Figure 2. HORMAD1 acts downstream of checkpoint signaling

(A) Indicated H1395 cells were exposed to PBS or HU for 4 hours, followed by subcellular fractionation. The nuclear fraction was subjected to immunoblotting with the indicated antibodies (Left). Graphs represent the mean band intensity over at least three experiments (Right). Error bars represent SEM. P value calculated by Mann-Whitney t-test; n=3.
